# ‘’Not a Universal Effect’’: Polyunsaturated Fatty Acids and Cognition from Pregnancy to Adolescence—A Narrative Review and Nutritional Guidance for a Mediterranean Population

**DOI:** 10.3390/nu18193264

**Published:** 2026-10-03

**Authors:** Elissar El-Hayek, Marguerita Akiki, Marie-Joelle Chbat, Rita Keyrouz, Celine Mrad, Marc Bouji, Yonna Sacre

**Affiliations:** 1 Department of Biology, Faculty of Arts and Sciences, Holy Spirit University of Kaslik (USEK), Jounieh P.O. Box 446, Lebanon; marguerita.akiki@net.usek.edu.lb (M.A.); mariejoelle.a.chbat@net.usek.edu.lb (M.-J.C.); rita.keyrouz@net.usek.edu.lb (R.K.); 2 Department of Nutrition and Food Sciences, Faculty of Arts and Sciences, Holy Spirit University of Kaslik (USEK), Jounieh P.O. Box 446, Lebanon; celine.n.mrad@net.usek.edu.lb; 3 Laboratory of Cognitive and Molecular Neuroscience, Faculty of Sciences, Saint Joseph University of Beirut (USJ), North Campus, Tripoli P.O. Box 44, Lebanon; marc.bouji@usj.edu.lb

**Keywords:** adolescents, children, DHA, EPA, FADS genetic polymorphisms, fatty acid metabolism, gene–diet interaction, Mediterranean diet, monounsaturated fatty acids, pregnancy

## Abstract

Polyunsaturated fatty acids (PUFAs), particularly docosahexaenoic acid (DHA) and eicosapentaenoic acid (EPA), are an important class of bioactive lipid mediators, essential for brain development from gestation through adolescence. Yet human studies evaluating the benefits of PUFAs for cognition remain inconsistent. This narrative review synthesizes studies from PubMed, Scopus, and Google Scholar examining PUFA intake, status, and supplementation in relation to cognitive and neurodevelopmental outcomes. It uses a thematic approach rather than the systematic search and screening protocol of a systematic review. This review also discusses the genetic determinants of PUFA metabolism and their association with neurodevelopmental outcomes, in addition to nutritional recommendations for Lebanon, a Mediterranean country. Animal models consistently show neuroprotective and anti-inflammatory effects of omega-3 PUFAs during critical windows of brain development. However, evidence from human studies evaluating the cognitive benefits of PUFAs remains limited and inconsistent, with more pronounced benefits in specific populations with low baseline PUFA status, learning difficulties, ADHD, or preterm birth. Genetic variation in genes involved in PUFA metabolism such as FADS1, FADS2, and ELOVL2/5 affects the capacity to synthesize long-chain PUFAs from dietary precursors, helping explain variability in response to omega-3 supplementation. Lebanon’s traditional Mediterranean diet already supplies PUFAs and MUFAs; the real challenge is improving adherence to it through nutrition education and policy.

## 1. Introduction

During pregnancy, toddlerhood, and adolescence, adhering to a healthy diet is essential to supporting optimal growth, brain development, and long-term health outcomes. PUFAs, especially DHA and AA, make up 20–25% of brain fatty acids and are crucial for myelination, neuronal development, and visual function during early life [1,2]. During the first trimester of pregnancy, DHA accumulates significantly in fetal brain and tissues. Adequate maternal dietary intake of PUFAs during pregnancy directly influences DHA availability [3]. Moreover, high intakes of long-chain *n*-3 PUFAs in toddlers and young children have been linked to beneficial effects on cognition, behavior, respiratory health, and cardiovascular risk factors. This can be explained by the fact that DHA accumulation in nervous tissue increases 30-fold during the first two years of life [4]. Insufficient intakes of PUFAs during these critical periods of growth and development may increase the risk of developing chronic diseases later in life [5].

Adequate MUFA intakes have been associated with reduced cardiovascular disease risk, improved serum lipid profiles, and anti-inflammatory effects [6]. Maternal plasma MUFA levels during pregnancy, particularly oleic acid, have been inversely associated with inflammatory biomarkers and gestational diabetes risk, underscoring their relevance to both maternal health and fetal growth [7]. In contrast, direct evidence linking MUFA intake to cognitive and neurodevelopmental outcomes remains very limited. PUFAs have been studied in relation to cognition far more extensively than MUFAs, as detailed in the upcoming sections.

The FAO recommends that total fat constitute 20–35% of total energy intake, with PUFAs contributing 6–11%, of which LA and ALA should constitute 2.5–9% and 0.5% of total energy intake, respectively [8]. EPA and DHA combined should reach 0.250–2 g/day [8]. MUFA intake is determined by difference and can reach up to 15–20% of total energy intake based on total fat intake [8]. According to EFSA, pregnant and lactating women should consume an additional 100–200 mg/day of DHA above the regular adult requirement of 250 mg/day of EPA and DHA in order to support retinal and fetal brain development [9]. For infants and toddlers up to 24 months, recommendations for DHA are 100 mg/day, increasing to 250 mg/day of EPA and DHA for older children and adolescents [9].

Among pregnant women, toddlers, and adolescents, studies from various countries have reported inadequate intakes of unsaturated fatty acids. In Canada, toddlers and pre-school children were found to consume inadequate amounts of total PUFA, total MUFA, *n*-6 PUFA, EPA, and DHA. However, the daily dietary intake of *n*-3 PUFA among toddlers was shown to be slightly above recommended levels [4,5]. Similarly, a study conducted in France showed that the daily intakes of *n*-3 and *n*-6 PUFAs, particularly LA, among children aged 3–10 years and adolescents aged 11–17 years were below the recommendations [10].

Among Indian pregnant women, the average daily intakes of ALA, EPA, and DHA were below the recommendations, and total MUFA and PUFA intakes were inadequate during the first, second, and third trimesters [1]. Mexican pregnant women also did not meet the recommended intakes for total MUFA, total PUFA, LA, ALA, EPA, and DHA [2]. Additionally, in New Zealand, pregnant women reported suboptimal intakes of long-chain *n*-3 PUFAs [3].

In European countries, including Belgium, France, Germany, Switzerland, the Netherlands, Nordic countries, Poland, and Spain, it was reported that the intakes of *n*-3 and *n*-6 PUFAs were below the recommendations in pregnant and lactating women, infants, children, and adolescents [11].

In Lebanon, a Mediterranean country, pregnant women reported inadequate intakes of MUFA and PUFA during all trimesters [12]. Evidence from Canada, France, India, Mexico, New Zealand, and several European countries shows a suboptimal consumption of unsaturated fatty acids. Similarly, findings in Lebanon align with these observations, especially among pregnant women.

As the Mediterranean diet is part of the Lebanese culture, vulnerable groups such as pregnant women, toddlers, and adolescents are theoretically exposed to a varied dietary pattern that is rich in MUFA and PUFA. Despite that, current intake levels of these groups are inadequate and tend to fall below the recommendations. This emphasizes the importance of adhering to the Mediterranean diet since its components are good sources of unsaturated fatty acids.

The findings presented above indicate that inadequate intakes of these fatty acids are widespread across critical periods of growth and development, and affect populations in the Eastern Mediterranean area, including Lebanon. Given their important biological roles, it is imperative to move beyond epidemiological observation and examine, in depth, the mechanistic pathways through which PUFAs exert their physiological effects. Understanding these mechanisms is necessary to explain why dietary adequacy matters and how deficiencies translate into tangible health risks across the lifespan. In this context, it is worth highlighting that Mediterranean populations hold a distinct dietary advantage, given that the Mediterranean diet is rich in both PUFA and MUFA. However, dietary intake alone does not fully determine an individual’s PUFA status: genetic variation in the enzymes that metabolize PUFAs also shapes how efficiently dietary precursors are converted into the long-chain forms relevant to brain health, and this variation differs across ethnic and ancestral populations, including those of Mediterranean origin. Understanding both dietary and genetic determinants of PUFA status is therefore essential to interpreting why PUFA supplementation shows inconsistent cognitive benefits across populations, and to formulating nutritional guidance appropriate for a specific population such as Lebanon.

## 2. Methodology

This narrative review was conducted through a literature search for studies examining the relationship between polyunsaturated fatty acids (PUFAs) and cognition, published between 2010 and 2025. We used PubMed, Scopus, and Google Scholar; Google Scholar served as a supplementary source to identify relevant publications that may not have been indexed in PubMed or Scopus.

For the parts related to animal models and human studies, the search strategy combined terms related to PUFAs (“polyunsaturated fatty acids,” “PUFA,” “omega-3,” “docosahexaenoic acid,” “DHA,” “eicosapentaenoic acid,” “EPA,” and “fish oil”) with terms related to cognition (“cognition,” “cognitive function,” “memory,” “learning,” “neurodevelopment,” and “brain”). Inclusion criteria for these parts comprised original research articles involving randomized controlled trials (RCTs), observational studies, experimental animal studies, and relevant meta-analyses written in English investigating the effects of PUFAs on cognitive function or brain health. A limited number of high-quality reviews were retained when they contributed mechanistic insight.

Animal studies using aged models of cognitive decline were excluded, as aging involves neurodegenerative processes (e.g., progressive neuronal loss, accumulated cellular senescence) that are mechanistically distinct from non-degenerative physiological processes of non-aged brains. However, a subset of animal studies using mature (non-aged) models was retained, even when the models themselves were not developmental, when they contributed mechanistic insight into PUFA-related neuroprotective, anti-inflammatory, or metabolic pathways. These pathways are mechanistically conserved across life stages, making findings from mature models relevant to understanding how PUFAs support brain health during development.

Human studies were excluded if they did not report cognitive or neurodevelopmental outcomes or if they were conducted only in adult populations without pediatric relevance.

For the part related to Ethnic and Interindividual Genetic Variability in PUFA Metabolism (Section 2.5), we combined the PUFA-related terms with genetic keywords, including “*FADS*,” “*FADS1*,” “*FADS2*,” “*ELOVL2*”, “*ELOVL5*”, “polymorphism,” “single nucleotide polymorphism (SNP),” “genotype,” “genetic variant,” “gene–diet interaction,” and “gene–nutrient interaction.” We included GWAS, candidate-gene association studies, birth cohort studies, and post hoc genetic sub-analyses of RCTs regardless of the age of the human participants studied. Because genetic variants are fixed across the lifespan, foundational gene–PUFA associations established in adult cohorts remain relevant to younger populations, and excluding them would limit the interpretation of later research. One foundational study, Tanaka et al. (2009) [13], was selected even though it was conducted before 2010, because it constitutes a reference point that several other subsequent studies have cited.

For the part related to Genetic Variation in PUFA-Related Genes and Cognitive Development (Section 2.6), we combined the PUFA- and genetic-related terms with cognition-related keywords. Studies were required to report a cognitive or neurodevelopmental outcome in a pediatric-relevant population. One reference study, Caspi et al. (2007) [14], was cited even though conducted before 2010, because it constitutes an important reference point, essential for the interpretation of subsequent studies.

**Figure 1:**
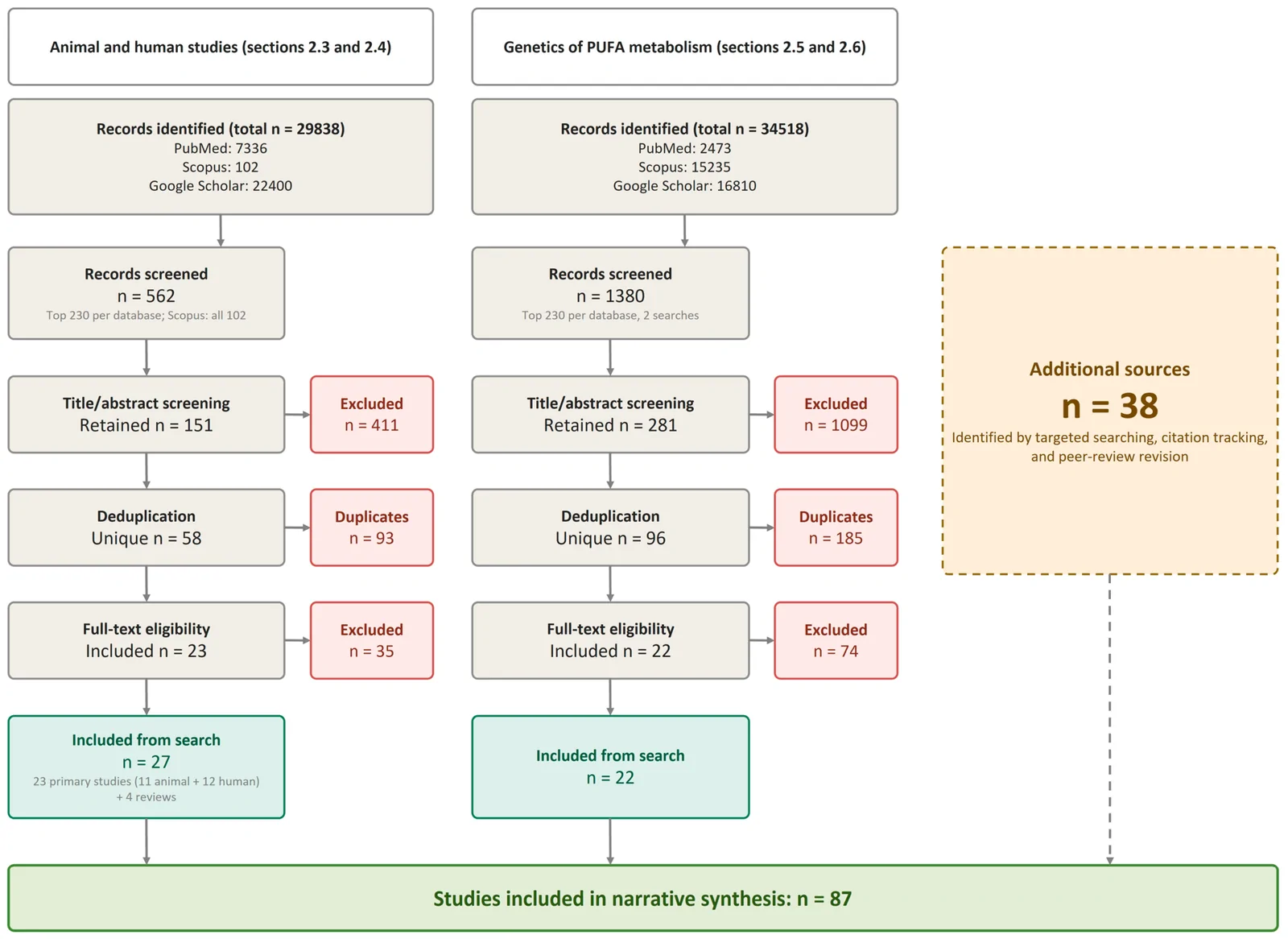
Flow chart of the study identification and selection process.

Overall, three searches were performed: one for the animal and human studies (Section 2.3 and Section 2.4) and two for the genetics sections (Section 2.5 and Section 2.6). For each search, results were sorted by relevance, and the first 230 records per database were screened (all records when fewer were returned). In total, 87 studies met these criteria and form the basis of this narrative synthesis. The study selection process is represented in Figure 1.

Searches were conducted in PubMed, Scopus, and Google Scholar (2010–2025) for two thematic areas: animal and human studies (Section 2.3 and Section 2.4) and genetics of PUFA metabolism (Section 2.5 and Section 2.6). Records identified correspond to the total number of results returned by each database. Because this is a narrative review, results were sorted by relevance and the first 230 records per database were screened; all records were screened when fewer than 230 were returned. One search was performed for the animal and human studies (Section 2.3 and Section 2.4) and two searches for the genetics Section 2.5 and Section 2.6. Duplicates were removed after title/abstract screening and before full-text assessment. Additional sources were identified through targeted searching, citation tracking and peer-review revision.

### 2.1. Objectives

The relationship between PUFAs and cognition is not straightforward: reported benefits are inconsistent across studies and appear to depend on the population studied, the timing and dose of exposure, and individual variation in PUFA metabolism. The present narrative review addresses these sources of variability, with particular focus on ethnic and interindividual genetic variability in the genes involved in PUFA metabolism and their relation to cognitive and neurodevelopmental outcomes, before closing with nutritional recommendations for Lebanon and other Mediterranean populations. Concretely, This review has four objectives: (1) to summarize mechanistic and animal-model evidence linking PUFA intake/status/supplementation to brain function, including neuroprotective, anti-inflammatory, and metabolic pathways relevant to cognition, across both developmental and mature (non-aged) models; (2) to synthesize human trials and cohort studies investigating PUFA intake/status/supplementation in relation to cognitive and neurodevelopmental outcomes from pregnancy through adolescence; (3) to examine the genetic determinants of PUFA metabolism, including variants in *FADS1*, *FADS2*, and *ELOVL2/5*, and their influence on individual and ethnic variability in response to PUFA intake/status/supplementation; and (4) to translate this evidence into practical, culturally grounded nutritional recommendations for Lebanon and other Mediterranean populations. MUFA intake is addressed throughout as a co-occurring component of the same dietary patterns, given its abundance in the Mediterranean diet. However, since the preclinical and clinical evidence directly linking MUFA to cognitive outcomes remains considerably less developed than that for PUFA, the sections addressing mechanistic evidence in animal models, human studies, and genetic studies will focus on PUFAs rather than MUFAs. The review proceeds accordingly, moving from mechanistic and preclinical evidence, to human cognitive and neurodevelopmental outcomes, to the genetic modifiers of individual response, before closing with dietary implications, encompassing both PUFA and MUFA, for a Mediterranean, and specifically Lebanese, context.

### 2.2. Structure, Classification, and Biological Roles of PUFAs

PUFAs are an important class of biologically active fatty acids [15]. The hydrocarbon chain contains two or more cis, methylene-interrupted double bonds [15]. This structural feature differentiates PUFAs from saturated and monounsaturated fatty acids and is the basis for the physicochemical properties relevant to their biological function [16,17]. Variations in position, number, and geometry of these double bonds influence membrane packing, lipid–protein interactions and membrane fluidity, thereby affecting membrane architecture, cell signaling, and numerous cellular functions [16,17].

There are two families of PUFAs, classified based on the position of the first double bond relative to the terminal methyl end of the carbon chain: the omega-3 (*n*-3) and omega-6 (*n*-6). The omega-3 family includes α-linolenic acid (ALA), eicosapentaenoic acid (EPA), and docosahexaenoic acid (DHA). The omega-6 family includes linoleic acid (LA), arachidonic acid (AA), and γ-linolenic acid (GLA). This classification corresponds not only to structural differences but also to distinct metabolic pathways and biological functions [15,16].

Polyunsaturated fatty acids are now recognized as essential dietary components [18]. Adequate PUFA intake is essential for optimal physiological function [19]. Several studies have linked omega-3 PUFAs to improved attention, cognitive performance, visual acuity, and behavioral outcomes [18,20,21].

At the cellular level, PUFAs constitute a component of phospholipid bilayers and are found within the phosphatidylcholine and phosphatidylethanolamine fractions of the membrane. Enrichment of membranes with long-chain PUFAs such as AA, EPA and DHA has been experimentally shown to affect membrane permeability, receptor clustering, and signal transduction efficiency. These effects result from the conformational flexibility conferred by the multiple cis double bonds, which reduces van der Waals interactions between lipid chains and increases lateral membrane dynamics [22,23].

PUFA, particularly DHA and AA, are present at high levels in the brain and play a key role in synaptogenesis and synapse function. Hence, deficiency in PUFA could lead to synaptic abnormalities affecting cognitive abilities and contributing to the pathogenesis of neurological disorders such as autism spectrum disorder, ADHD, schizophrenia and depression [24].

Beyond their structural roles, PUFAs serve as precursors for a diverse array of bioactive lipid mediators that regulate inflammation, immunity and cellular signaling. Both omega-6 and omega-3 PUFAs are enzymatically converted into eicosanoids, docosanoids, and related metabolites that exert potent autocrine and paracrine effects [25]. These lipid mediators participate in both pro-inflammatory and pro-resolving pathways [22,23,26]. Particularly in brain health, neuroinflammation, supported by AA-derived eicosanoids such as prostaglandins and leukotrienes, is a physiologic mechanism essential to protect from invading pathogen and remove damaged cells. However, resolution of the inflammation is essential to avoid excessive tissue injury and supporting cognition. AA and EPA compete for the same cyclooxygenase enzymes (COX), so increasing the EPA:AA ratio shifts eicosanoid production away from the highly active, AA-derived inflammatory mediators toward less inflammatory, EPA-derived counterparts [27,28]. Beyond this substrate competition, EPA and DHA also give rise to a distinct class of specialized pro-resolving mediators such as resolvins, protectins, and maresins that, in addition to lipoxins (lipid mediators resulting from the conversion of AA-derived eicosanoids) actively signal the termination of inflammation through dedicated receptors [27,28]. In practice, diets richer in EPA and DHA, favor the less inflammatory, pro-resolving pathways described above [29].

In addition to its role in neuroinflammation, PUFAs are also known to modulate oxidative stress. Through this mechanism, they may have protective effects against cognitive impairment psychiatric and behavioral disorders [30,31].

Although, numerous studies have reported a positive association between dietary intake of PUFAs and cognitive performance, the findings remain inconsistent among studies. This review provides a benchmark of the current literature on the relationship between PUFAs and cognition with a particular focus on children and adolescents. The first part of this review focuses on the association between PUFA intake and or PUFA levels in erythrocyte membranes and cognition in animal models and Humans from pregnancy through adolescence. The second part addresses the differences in PUFA metabolism across different populations, highlighting the importance of adjusting the PUFA intake recommendations based on ethnicity and genetic variations.

### 2.3. Mechanisms of Action of PUFAs in the Brain: Insights from Animal Models

A limited number of animal studies have demonstrated the important roles of omega-3 polyunsaturated fatty acids (PUFAs), particularly docosahexaenoic acid (DHA) and eicosapentaenoic acid (EPA), in supporting cognitive development and neural function under different forms of stress. In a mouse model of perinatal DHA deficiency, leading to more than 50% reduction in brain DHA, long-lasting impairments in spatial memory and downregulation of key hippocampal synaptic proteins have been observed. When offspring were switched to a DHA-adequate diet at 3 weeks, 2 months, or 4 months of age, brain DHA levels were restored at every switch age tested. However, spatial memory deficits were normalized only when the diet was switched at the earliest time point (3 weeks), indicating that DHA repletion alone is not sufficient to reverse cognitive impairment once a critical developmental window has closed [32]. Similarly, Fu et al. (2022) [33] showed that post-weaning supplementation with EPA-enriched ethanolamine plasmalogens effectively restored DHA levels in the brain and liver of mice depleted of DHA during early life.

Unfortunately, studies isolating the effects of PUFA supplementation in young, healthy animals under normal (non-deficient, non-pathological) conditions remain scarce in the literature. To further elucidate mechanistic pathways linking PUFAs to brain health, this review therefore also incorporated non-aged models subjected to other forms of pathological stress. Across these models, PUFA supplementation consistently supported neuroprotection (findings summarized in detail in Table 1). For instance, in seizure models, dietary enrichment with EPA or DHA significantly reduced neuroinflammation, preserved myelin integrity and enhanced antioxidant pathways [34]. In an animal model of prenatal methylmercury exposure, maternal DHA intake during gestation increased offspring brain DHA content and protected against motor and short-term memory impairments induced by this environmental neurotoxin [35]. In mice exposed to rotenone, a Parkinson causing neurotoxin, PUFA supplementation has improved motor coordination [36]. Interestingly, in a mouse model of diabetes-related cognitive dysfunction it has been reported that co-supplementation of fish oil with folate and vitamin B_12_ could interact with epigenetic mechanisms to improve cognition [37]. In a rat model of chronic stress, it was shown that induced brain oxidative damage can be reduced by the administration of DHA-Rich Fish Oil [38].

Moreover, preclinical studies using models of chemotherapy [29,39] and surgery-induced neuroinflammation [40] support a protective role for PUFAs against synaptic damage. Across these models, EPA and DHA supplementation consistently supported neuroprotection through different mechanisms such as preservation of synaptic scaffolding proteins, downregulation of neuroinflammatory and blood–brain barrier–disrupting genes, and suppression of microglial activation (findings summarized in detail in Table 1). Although these models are not developmental, they offer mechanistic insight into how PUFAs modulate neuroinflammatory pathways that may also be relevant to brain health during development.

Collectively, these animal models demonstrate that omega-3 PUFAs support brain health through neuroprotective, anti-inflammatory, antioxidant and metabolic pathways across different experimental conditions. Two studies suggest that PUFA deficiency during early neurodevelopment can produce effects that later repletion does not fully reverse, pointing to this period as one of heightened vulnerability. Notably, none of the reviewed studies examined whether PUFA supplementation improves cognition in young animals under normal conditions, raising the question of whether PUFAs confer benefits in the young, healthy brain. This represents a gap consistent with the broader literature, where most evidence of cognitive benefit comes from animals that were previously deficient, aged, or subjected to pathological conditions. The next section of this review will discuss the cognitive benefits in human pediatric populations.

**Table 1:** Animal Studies Studying Omega-3 PUFA Effects on Brain Function, Neuroinflammation, and Cognition.

Study (Author, Year)	Animal Model	Intervention	Cognitive/Behavioral Outcome	Mechanism	Limitations
Lozada et al., 2017 [32]	Mouse, perinatal DHA deficiency (>50% brain DHA reduction); pregnant mice *n* = 3/group; offspring *n*= 8–19/group	Switched to ω-3-adequate diet at 3 weeks, 2 months, or 4 months	Spatial learning/memory deficits at 6 months; only 3-wk switch normalized cognition, despite DHA restoration at all switch ages	Critical window: synaptic protein deficits corrected only with earliest diet switch, despite DHA recovery at all ages	Small breeding cohort
Fu et al., 2022 [33]	Mouse, post-weaning; *n* = 8/group	EPA-enriched ethanolamine plasmalogen supplementation	Not assessed	Restored/increased DHA levels in brain and liver	Biochemical endpoint only
Yang et al., 2023, a [34]	Mouse, young, chemically induced seizures; *n*= 10/group	Dietary EPA or DHA enrichment (pre-seizure)	Improved seizure-related behavioral outcomes; EPA showed superior neuroprotection compared to DHA	Reduced neuroinflammation; preserved myelin integrity; enhanced antioxidant pathways	Primary outcome measured was depressive symptoms; no direct cognitive testing performed
Oguro et al., 2023 [35]	Mouse, prenatal methylmercury exposure; Pregnant mice *n*= 4–5/group; offspring *n* = 3–10/group	Maternal DHA intake during gestation	Protection against methylmercury-induced motor and short-term memory impairments	Increased offspring brain DHA content; reduced oxidative stress; preserved neurons	Only tested in the context of methylmercury exposure; effects of maternal DHA supplementation in a normal (non-toxicant-exposed) pregnancy were not examined
Alves et al., 2024 [36]	Mouse, rotenone-induced Parkinsonism; *n*= 10/group	Fish oil ± curcumin	Improved motor coordination	Fish oil alone increased lipid peroxidation; curcumin co-administration added antioxidant protection	Chemically induced Parkinsonism model, not developmental in nature; motor outcome only, no cognitive measure reported.
Chen et al., 2025 [37]	Mouse, diabetes-related cognitive dysfunction; *n* = 6/group	Fish oil + folate + vitamin B_12_	Synergistic improvement in cognitive abilities	Restored DNA methylation balance	Non-developmental model; combined supplementation, does not isolate the effect of omega-3 PUFA alone
Yildiz and Medina, 2024 [41]	Gray mouse lemur; *n* is not reported (reanalysis of published means)	Chronic fish oil supplementation	Not assessed	Increased efficiency of glucose metabolism across brain regions (thermodynamic modeling)	Secondary analysis of previously published data
Orchard et al., 2018 [29]	Ovariectomized female mice; doxorubicin-based chemotherapy; *n* = 11–14/group	Low-sucrose diet with EPA + DHA supplementation	Not assessed	Preserved expression of Shank 3 (synaptic scaffolding protein essential for neuronal structure/communication); reduced cortical inflammatory cytokines IL-5, IL-6, and KC/GRO (a rodent chemokine that recruits immune cells during inflammation)	Non-developmental model; molecular endpoints only
Ormiston et al., 2025 [39]	Ovariectomized female mice; doxorubicin-based chemotherapy, *n* ~7/group	High-fat (HF), high-fat + EPA/DHA (HF*n*-3), or low-fat (LF) diet, all with chemotherapy	Not assessed	HF diet + chemotherapy upregulated neuroinflammation/neurodegeneration genes vs. LF; HF*n*-3 diet attenuated this by downregulating Mapkapk2, Aqp4, S100b (neuroinflammation/BBB regulation)	Non-developmental model; molecular endpoints only
Yang et al., 2023, b [40]	Mice undergoing orthopedic surgery; *n* = 4–8/group	Prophylactic intravenous omega-3 fatty acid emulsion	Reduced postoperative delirium-like behavior, with better attentional performance	Attenuated microglial activation; preserved blood–brain barrier integrity, partly through protection of pericytes and suppression of perivascular macrophage activation	Non-developmental model
Asari et al., 2022 [38]	Rat, chronic stress; *n* = 10/group	DHA-rich fish oil	Reduced chronic stress-induced brain oxidative damage	Decreased serum corticosterone levels; reduced brain lipid peroxidation	Non-developmental model; biochemical endpoints only

Abbreviations: BBB, blood–brain barrier; DHA, docosahexaenoic acid; EPA, eicosapentaenoic acid; HF, high-fat diet; HF*n*-3, high-fat diet enriched with EPA/DHA; IL-5, interleukin-5; IL-6, interleuki*n*-6; KC/GRO, keratinocyte chemoattractant/growth-regulated oncogene; LF, low-fat diet; Mapkapk2, mitogen-activated protein kinase-activated protein kinase 2; S100b, S100 calcium-binding protein B; ω-3, omega-3.

### 2.4. PUFA and Cognition in Children and Adolescents: What the Human Studies Tell Us

The effect of omega-3 PUFAs on cognitive development in children and adolescents has been the focus of numerous studies over the past two decades. Findings, although heterogeneous, collectively suggest that PUFA status is linked to cognitive and behavioral outcomes across different developmental stages and populations. Evidence from clinical trials, cohort studies, and meta-analyses highlights both the potential and the limitations of PUFA supplementation for cognitive function in youth.

Several studies have demonstrated that omega-3 supplementation is associated with improved attention and behavioral outcomes, particularly in children with attention-deficit/hyperactivity disorder (ADHD).

In a randomized controlled trial (RCT), Milte et al. (2012) [42] have shown no significant effect of EPA and DHA supplementation on literacy, cognition and behavior in Australian children with ADHD. However, increased erythrocyte DHA after 4 months of supplementation was correlated with improved word reading and oppositional behavior. Similar effects were observed in another 12 weeks RCT comparing the effects of high-dose EPA and placebo on cognitive function in Taiwanese children and adolescents with ADHD [43]. Interestingly, this study has highlighted a beneficial effect of EPA treatment on cognitive symptoms in ADHD youth only if they have a low baseline endogenous EPA level. However, EPA supplementation in youth with high EPA levels may have a negative effect on cognition, which is a strong argument for checking a patient’s blood PUFA profile before starting supplementation in ADHD. On the other hand, an RCT conducted by Matsudaira et al. (2015) [44], on UK adolescent ADHD males have shown no effect of 12 weeks omega-3 and omega-6 supplementation on ADHD symptoms [44].

The effects of PUFA supplementation on cognition in healthy populations are even more variable. A cross-sectional study in typically developing Spanish adolescents found that higher red blood cell DHA levels were associated with better attention [21]. However, a neuroimaging study conducted by Morandini et al. (2022) [45] found no association between dietary omega-3 intake and vigilant attention (a cognitive ability involved in redundant or intellectually unchallenging tasks) in neurotypical children and adolescents from the Healthy Brain Network databank [45]. Similarly, the DOLAB study found that DHA supplementation did not significantly improve reading or working memory in the full cohort of UK underperforming readers, although a pre-specified subgroup with the lowest baseline reading scores showed significant improvement [46].

A trial conducted on Thai children found neurophysiological improvements with fish oil (DHA) supplementation—such as increased ERP (Event-Related Potential) amplitudes (reflecting stronger brain responses to cognitive tasks) but these changes did not consistently translate into observable behavioral gains [47]. In Omani children, fish oil supplementation outperformed a fish meal on one measure of executive function [48].

Collectively, these observations in ADHD and healthy youth highlight three important factors to be taken into consideration when assessing the efficacy of PUFA supplementation on cognition and behavior: the baseline nutritional status (in other words, children with lower DHA levels may benefit more); the cognitive vulnerability (meaning that youth with learning difficulties may show greater responsiveness), and the sensitivity of the measured outcome (e.g., ERP measures may capture small neural changes that behavioral ratings fail to detect).

Researchers have also examined how maternal omega-3 status and supplementation during pregnancy and lactation may influence neurodevelopment in infants and children. The ECLIPSES cohort study, conducted in Spain, revealed that maternal omega-6/omega-3 and ARA/DHA ratios in the third trimester were negatively associated with motor development in infants [49]. No significant correlation was observed during the first trimester [49]. These findings highlight the importance of optimizing the balance of fatty acids for appropriate fetal neurodevelopment. A randomized trial in Iran testing a fixed low dose of omega-3 supplementation (without considering omega-6 intake), during the entire pregnancy and for 30 days postpartum, showed no significant benefit on child neurodevelopment or growth [50]. These observations serve as a reminder that PUFA dose, timing of supplementation in addition to omega-6/omega-3 ratio should be taken into consideration when assessing its benefits on neurodevelopment and cognition.

In the same context, an RCT of high-dose maternal DHA supplementation reported no overall improvement in neurodevelopmental outcomes in preterm infants at 18 to 22 months, although a subgroup delivered before 27 weeks’ gestation showed potential language-related benefits [51]. This finding may be partly explained by variation in baseline PUFA status by gestational age. Indeed, preterm infants show significantly lower DHA and ARA levels than term infants, correlating with gestational age [52]. These low levels can be explained by the fact that premature infants have been exposed to a shorter period of placental fatty acid transfer, combined probably with their immature capacity to synthesize endogenous fatty acids.

Taken together, of the human studies summarized in Table 2: one (Pinar-Martí et al., 2023 [21]) reported a significant positive association across the whole sample between PUFA and cognitive performance; six (Milte et al., 2012 [42]; Chang et al., 2019 [43]; Richardson et al., 2012 [46]; Sittiprapaporn et al., 2022 [47]; Al-Ghannami et al., 2019 [48]; Guillot et al., 2022 [51]) reported associations restricted to a specific subgroup or outcome domain; one study (Shahabi et al., 2025 [49]) found a significant adverse association, restricted to the third trimester of pregnancy, between elevated maternal *n*-6/*n*-3 and ARA/DHA ratios and infant motor development. This heterogeneity in the findings may be partly explained by methodological differences across studies. Dosing varied considerably, from low-dose (Sarrafi et al. 2024 [50]) to high-dose interventions (Chang et al. 2019 [43] and Guillot et al., 2022 [51]); notably, Sittiprapaporn et al., 2022 [47] directly compared low- and high-dose DHA within the same trial and found a dose–response relationship for neurophysiological (ERP) measures but not for behavioral outcomes. Duration and timing of supplementation also differed, ranging from 12-week postnatal trials in school-aged children (Chang et al., 2019 [43]; Matsudaira et al., 2015 [44]) to prenatal supplementation throughout gestation (Guillot et al., 2022 [51]). In the latter study, benefit emerged only in infants born before 27 weeks, suggesting that the developmental window of exposure may be a key determinant of treatment response. Finally, the assessment tools ranged from standardized cognitive and behavioral scales to neurophysiological measures such as ERP amplitude, and from RCTs to cross-sectional or observational design. All these methodological differences combined with biological factors (such as baseline PUFAs status, cognitive vulnerability, and genetic or ethnic variability in PUFA metabolism), make it difficult to draw a unified conclusion about the relation between PUFAs and cognition in youth. Systematic reviews and meta-analyses offer a more comprehensive perspective, suggesting that while omega-3 supplementation shows modest benefits in certain domains such as attention or memory, the overall effect sizes are small and context-dependent. Several factors such as genetic polymorphisms, co-nutrient status, and environmental influences may contribute to this variability. For example, Basak et al. (2021) [53] emphasize the need for both DHA and ARA to optimize neurodevelopment, while Zinkow et al. (2024) [54] describe potential interactions between DHA and the gut microbiome that influence brain function. Other reviews argue that the benefits of omega-3 may be more pronounced in children with nutritional deficiencies or developmental risks, rather than in well-nourished, healthy populations [55,56].

This body of evidence suggests that PUFAs support cognitive and behavioral development in children, though the benefit is far from universal and varies according to timing, dose and population characteristics in addition to other factors related to the study design. While the reviewed trials did not report significant adverse effects [42,43,44,46,47,48,50,51], and supplementation may offer targeted benefits in the specific groups identified, its widespread use in general pediatric populations should be considered with realistic expectations. Future research should aim to clarify optimal dosing, timing, and combinations with other nutrients, and to identify the populations most likely to benefit from the supplementation.

Because genetic and ethnic variability in PUFA metabolism is an important explanation proposed for these population-level differences, the following sections examine this variability in detail, starting from the enzymatic pathways that convert dietary precursors into long-chain PUFAs and extending to how differences in these pathways may relate to cognitive and developmental outcomes.

### 2.5. Variability in PUFA Metabolism and Inconsistent Cognitive Effects: The Role of Genetic and Ethnic Differences

#### 2.5.1. Enzymes Involved in the Metabolism of PUFAs

Among the polyunsaturated fatty acids mentioned, only LA and ALA are essential [25], because humans lack the enzyme necessary to introduce double bonds beyond the Δ9 position of the fatty acid chain [57]. LA and ALA are metabolized into longer, more unsaturated PUFAs such as EPA, DHA and AA through desaturation and elongation pathways. These pathways are catalyzed by two main families of enzymes: The fatty acid desaturase (FADS) and Elongation of very long-chain fatty acids (ELOVL) [58].

Humans have three *FADS* genes (FADS1, 2 and 3), located in a gene cluster on the long arm of chromosome 11 (11q12 –q13.1) [13]. Fatty acid desaturase 1 (FADS1), also known as D5D, encodes ∆-5 desaturase that is responsible for catalyzing desaturation of 20-carbon (C20) PUFA. It is a rate limiting enzyme that converts dihomo-gamma-linolenic acid (DGLA) to AA and eicosatetraenoic acid (ETA) to EPA [59]. In contrast, fatty acid desaturase 2 (FADS2) exhibits a broader activity by catalyzing delta six, delta four, and delta eight desaturation by acting on 18-carbon (C18), 20-carbon (C20), and 22-carbon (C22) substrates [60]. It initiates the PUFA biosynthetic pathway and provides the final desaturation step required for the production of docosahexaenoic acid (DHA) [61]. The role of FADS3 is less known compared to FADS1/2.

In tandem with FADS enzymes, elongase (ELOVL) enzymes play an equally important role in the biosynthesis of PUFA. Elongases are a family of 7 enzymes that are responsible for elongation of the shorter precursor essential fatty acids, among which ELOVL2 and ELOVL5 are PUFA-specific [62]. Unlike the clustered *FADS* genes, *ELOVL 2* and *ELOVL 5* are found in distinct regions on chromosome 6. Specifically, *ELOVL5* is situated on the short arm at 6p12.1, while *ELOVL2* is located further toward the telomere at 6p24.2 [63]. In terms of function, ELOVL5 catalyzes the elongation of 18-carbon (C18) and 20-carbon (C20) PUFAs, converting LA and ALA into their longer-chain metabolites. On the other hand, ELOVL2 shows a strong preference for 20-carbon (C20) and 22-carbon (C22) substrates. It is the rate-limiting enzyme required for the final elongation step in the biosynthesis of DHA (22:6*n*-3) [64].

These enzymes are critical in PUFA metabolism, each with distinct roles and substrate specificities. Their activities are influenced by genetic variations, which can have significant implications. Studies have demonstrated that genetic variation within PUFA metabolic genes contributes substantially to differences in PUFA profiles among different populations. Studies have identified single-nucleotide polymorphisms (SNPs) within *FADS1*, *FADS2*, and *ELOVL* genes that are associated with altered enzyme efficiency and shifts in circulating and tissue PUFA composition [65]. The following section explores how these variants differ across populations and ethnic groups.

#### 2.5.2. Ethnic and Interindividual Genetic Variability in PUFA Metabolism

By regulating PUFA metabolism, the *FADS* gene cluster plays an important role in numerous physio-pathologic processes such as risk of cardiovascular diseases, eczema, asthma, and cognition [66]. Multiple genome-wide and candidate gene association studies has shown that rs174537 (a single nucleotide polymorphism located near the *FADS1* and *FADS2* gene) is the strongest genetic determinant of AA serum levels [13,67]. At the rs 174537, the G allele is the ancestral (wild type allele) and the T allele is the derived allele [68]. The G allele is almost fixed in Sub-Saharan African populations (e.g., Kenya, Nigeria) [68]. Unlike Africans, Europeans carry a mixture of GG, GT and TT genotypes. They exhibit a North–South Gradient, the frequency of the G allele is nowadays higher in Northern Europe and lower in Mediterranean populations [69].

G allele at the rs174537 is associated with increased activity of delta-6 desaturase (D6D, encoded by *FADS2*) and delta-5 desaturase (D5D, encoded by *FADS1*), making the desaturation pathway far more efficient at converting the dietary omega-6 linoleic acid (LA) into the long-chain Highly Unsaturated Fatty Acids (HUFA), especially arachidonic acid (AA) [68]. It results in markedly higher AA: LA ratios [70,71], knowing that AA is a precursor of bioactive eicosanoids involved in inflammatory signaling [72].

African American participants in the VITAL *n*-3 HUFA trial demonstrated greater benefit from omega-3 supplementation than other ancestry groups. Moreover, the predominance of LA (>90% of total PUFA intake) in Western diets may disproportionately increase *n*-6 HUFA levels in individuals of African ancestry, warranting tailored dietary guidance (e.g., lower LA intake and higher EPA/DHA intake) [73]. Individuals of Northern European ancestry (e.g., Scandinavians) tend to have a lower AA:LA ratio and a modestly reduced capacity to synthesize long-chain *n*-6 HUFAs, whereas individuals of Southern European (Mediterranean) ancestry exhibit slightly greater conversion efficiency, which may modulate inflammatory and cardiovascular risk profiles and influence responsiveness to *n*-3 PUFA supplementation [69].

In addition to rs174537, Genome wide association studies (GWAS) identified multiple SNPs across the *FADS* cluster that may influence the conversion efficiency of LA to long-chain *n*-6 HUFA such as AA [74]. An increased concentration of omega-6 PUFAs (except DGLA) has consistently been associated with the major allele of the following SNPs in a homogeneous islander population [74]: rs174545, rs174546, rs174553, rs174556, rs174561, rs174568, rs99780, rs174570, rs174575, rs2524299, rs174583, rs498793, rs174611, rs174627 and rs1000778. It is important to note that these SNPs reside within a block of tight linkage disequilibrium (LD) spanning the *FADS* gene cluster. Because of this high genomic proximity, they are almost always co-inherited as a single block, or haplotype, collectively defining an individual’s metabolic efficiency [74].

Extending this genomic framework, the intronic *FADS1* variant rs174547 acts in strong linkage disequilibrium alongside the genetic panel revealed by Mathias et al. (2010), in the same homogeneous islander population mentioned earlier [74]. In fact, a Genome-Wide Association Study Conducted in an elderly Mediterranean population with metabolic syndrome, has shown rs174547 is a major genetic determinant of serum omega-3 PUFA, with the minor C allele being associated with lower omega-3 levels, independently of lifestyle variables or adherence to a Mediterranean dietary pattern [75].

In a cohort of Danish infants, polymorphisms within the *FADS* gene cluster were significantly associated with erythrocyte DHA levels at 9 months of age. Homozygous carriers of the minor allele of rs1535 had higher DHA levels, whereas carriers of the minor alleles of rs174448 and rs174575 had significantly lower DHA concentrations. These findings suggest that genetic variation in *FADS* contributes to inter-individual differences in DHA status during infancy, although, in this study, breastfeeding remained the strongest determinant of DHA levels [76].

In the same context, a meta-analysis of ten studies showed that the *FADS2* rs174575 minor G allele is associated with increased DGLA and decreased AA levels [77]. Subgroup analyses in this research revealed that these associations are particularly evident in European populations and in breast milk [77]. These findings suggest that *FADS* polymorphisms may influence infant PUFA status both indirectly, by modifying the LC-PUFA composition of breast milk, and directly, by affecting the infant’s own metabolic capacity to synthesize and process these fatty acids. These dual effects may contribute to inter-individual differences in PUFA profiles during early development.

It is important to mention that genetic factors could interact with epigenetic factors to shape the metabolism of PUFA. For instance, the rs174537 variant, already discussed above, is also associated with DNA methylation at the *FADS2* promoter, which in turn correlates with circulating omega-6 PUFA levels [78]. Diet is also known to directly alter this methylation. In fact, it has been shown in a human supplementation trial, that *n*-3 PUFA intake is able to change DNA methylation at the *FADS2* and *ELOVL5* promoters compared to olive oil [79]. Together, these findings suggest that PUFA metabolic phenotype depends on both fixed genetic variation and diet-modifiable epigenetic changes.

The next section focuses on whether the genetic variation already discussed translates into differences in cognitive and neurodevelopmental outcomes.

### 2.6. Genetic Variation in PUFA-Related Genes and Cognitive Development

Limited research has investigated the relationship between the genetic variation in the *FADS* gene cluster, PUFA status, and the potential long-term effects on neurodevelopmental outcomes. In the SKOT cohort at 36 months of age, *FADS* tag-SNPs previously associated with infant erythrocyte DHA levels did not show overall associations with behavioral outcomes. However, significant sex-specific interactions were observed, whereby alleles associated with higher DHA status were linked to improved cognitive scores in boys but lower scores in girls. No association was observed between concurrent erythrocyte DHA and behavioral measures, suggesting that early-life PUFA metabolism, rather than later DHA status, may play a role in neurodevelopmental programming. These findings indicate that the relationship between *FADS* polymorphisms, DHA availability, and cognitive outcomes is complex and may be modified by sex and interacting genetic pathways [80].

Early candidate-gene studies suggested that rs174575, may moderate the association between breastfeeding and cognitive development. Across two independent birth cohorts (from New Zealand and England), the authors found that breastfeeding was associated with higher IQ only in children carrying the rs174575 C allele (major allele), while GG homozygotes showed no IQ benefit from breastfeeding [14]. A study conducted in two Spanish birth cohorts demonstrated that maternal variants in the *FADS* gene cluster influenced LC-PUFA ratios in colostrum, which in turn were associated with higher cognitive scores in early childhood [81]. In addition, this study found that the absence of breastfeeding was associated with lower cognitive scores, particularly among children homozygous for the rs174468 GG genotype (*FADS1*) and the rs2397142 CC genotype (*ELOVL5*), pointing to a child-genotype × feeding-method interaction rather than a purely maternal effect [81].

However, subsequent research has reported inconsistent replication of this gene-environment interaction [82,83]. A recent large-scale analysis in the UK Biobank (2024) involving over 300,000 individuals found no evidence of interaction between *FADS2* genetic variants and self-reported breastfeeding status on cognitive outcomes [84].

On another hand, a post hoc analysis of the POSGRAD randomized controlled trial [85] examined whether maternal variants in the *FADS2* gene modify the effect of prenatal DHA supplementation on child neurodevelopment at 5 years. Pregnant women received either 400 mg/day algal DHA or placebo from mid-gestation until delivery, and offspring cognitive outcomes were assessed. The study focused on maternal *FADS2* SNPs rs174602 and rs174575. The results showed a significant gene–nutrient interaction for rs174602, where children of mothers homozygous for the minor allele (TT genotype) in the DHA supplementation group had higher cognitive scores, particularly in quantitative and memory domains, compared to controls. No consistent overall effect of DHA was observed across all genotypes. These findings suggest that the neurodevelopmental effects of prenatal DHA supplementation may depend, in part, on maternal *FADS2* genotype.

Evidence on the effects of gene polymorphisms on PUFA metabolism in the Middle East and North African (MENA) region remains limited. This gap highlights the need for more population-based genetic research in MENA populations.

Overall, current evidence suggests that the effects of PUFAs on cognition are both population- and individual-specific, partly driven by genetic variability in genes involved in PUFA metabolism. This variability may partly explain the inconsistent findings regarding the cognitive benefits of PUFA supplementation and underscores the importance of conducting studies in underrepresented populations, including those in the MENA region.

Despite this variability, ensuring adequate PUFA and MUFA intake remains a practical, population-level strategy for supporting cognitive development. The next section focuses on how Lebanon’s Mediterranean dietary tradition offers a natural advantage for meeting these PUFA and MUFA requirements.

### 2.7. Nutritional Recommendations: Leveraging the Mediterranean Diet to Optimize PUFA and MUFA Intake in the Lebanese Population

Although Lebanon has a Mediterranean dietary culture, inadequate intakes of PUFAs and MUFAs are prevalent among Lebanese pregnant women, toddlers, and adolescents. This gap between dietary potential and actual intake represents a public health challenge and a unique opportunity. In nutritional terms, the Lebanese population is fortunate because their traditional dietary pattern is abundant in MUFAs through the ample use of extra virgin olive oil, and in PUFAs through frequent consumption of fatty fish, walnuts, flaxseeds, and legumes. In Lebanon, these foods are readily accessible, culturally and traditionally familiar, and a large part of Lebanese cooking [12]. Nevertheless, it was revealed by national dietary surveys that there is a gradual transition to Western dietary patterns, particularly among children and adolescents, defined by a high consumption of ultra-processed, low nutrient, and high saturated fat foods. Sequential information from a Lebanon project showed that as low as 14–20% of adolescents in Lebanon will continue to adhere to a Mediterranean dietary pattern by 2030 [86]. This might lead to significant consequences for MUFA and PUFA sufficiency during critical neurocognitive and cardiovascular developmental periods.

Considering this, the subsequent recommendations are presented to encourage the Lebanese population, especially pregnant women, toddlers, and adolescents, to emphasize the widespread and natural sources of MUFA and PUFA foods that are already present in the Mediterranean dietary traditions. Firstly, at all stages of life, olive oil should be the main source of dietary fat, and it should replace butter, margarine, and vegetable shortening. A large prospective cohort study conducted in Spain verifies that maternal adherence to a Mediterranean diet complemented by olive oil and nuts from early pregnancy is linked to health benefits in children up to six years of age, including reduced rates of atopic eczema and bronchiolitis. Firstly, although nowadays olive oil and nuts are easily accessible for Lebanese women, their intakes are below the recommendations; thus, they should receive individualized dietary counseling placing importance on these foods as essential and non-restrictive elements of a healthy gestational diet [12]. Secondly, the consumption of oily fish should be increased to at least two to three servings per week across all vulnerable groups. For instance, fish such as sardines, mackerel, and salmon are among the most bioavailable dietary sources of the long-chain omega-3 PUFAs EPA and DHA, which have essential roles in fetal brain development, myelination, and neurocognitive function [1,4]. Additionally, inadequate DHA and EPA levels have been documented among Lebanese pregnant women during all trimesters; hence, practical nutrition education should be prioritized in this group, while focusing on affordable small fatty fish that are widely available [12]. Thirdly, the consumption of mixed nuts should be encouraged from toddlerhood onward to support the endogenous synthesis of omega-3 and MUFA intake. In particular, walnuts, flaxseeds, and chia seeds should be encouraged, and they are consistent with Lebanese cuisine, while also being affordable and sustainable. Fourthly, from a public health perspective, the persistent economic crisis and nutrition shift in Lebanon emphasize the importance of establishing and implementing food-based dietary guidelines that are affordable, culturally appropriate, and customized to the needs of vulnerable groups [87]. These guidelines should encourage the return to the traditional Lebanese Mediterranean diet as an evidence-based and practical dietary pattern that can help meet PUFA and MUFA requirements throughout different stages of life. School-based nutrition programs, prenatal dietary counseling, and community-level food policy interventions should emphasize the Mediterranean diet as a realistic and accessible eating pattern. Since Lebanon is part of the Mediterranean region and shares similar cultural practices, dietary patterns, foods, and ingredients with other Mediterranean countries, dietary recommendations for the Lebanese population can also be applied more broadly across the region. For example, the consumption of olive oil, nuts, and oily fish should be increased, as these foods are available and accessible throughout the Mediterranean. Mediterranean countries, including Lebanon, are privileged to have dietary patterns rich in MUFA and PUFA, in contrast to other countries where diets are predominantly high in saturated fats and ultra-processed foods.

## 3. Limitations

This narrative review has several limitations. First, the search was restricted to studies published in English; therefore, relevant literature published in other languages may have been missed. Second, the search and screening process did not follow a systematic, PRISMA-based protocol; as such, some relevant studies may not have been captured, and the possibility of selection bias cannot be excluded. Third, because few developmental animal studies were available, studies using mature (non-aged) models were also included to provide mechanistic insight; this may limit the direct developmental relevance of some findings summarized in Table 1. Fourth, the human studies reviewed did not follow a consistent methodology, varying in the tools and scales used to assess cognition and neurodevelopment, sample size, and the type and dose of PUFA supplementation used, which limits direct comparability across studies. Fifth, the human cohort and cross-sectional studies cited may not have adjusted for the same confounders (e.g., socioeconomic status, maternal IQ, overall diet quality), which could affect the reported associations between PUFA status and cognition. Sixth, studies with negative or statistically nonsignificant findings are less likely to be published, which could bias the conclusions toward apparent benefits of PUFA supplementation. Finally, this review’s search concluded in 2025, so more recent GWAS and RCT findings are not captured and may alter the conclusions.

## 4. Conclusions

Taken together, the evidence examined in this review indicates that the relationship between PUFAs and cognitive development is not simple or universal, but is shaped by developmental timing, baseline nutritional status, and individual genetic variation in PUFA-metabolizing enzymes. Animal models subjected to different forms of stress consistently support neuroprotective and anti-inflammatory roles for omega-3 PUFAs, particularly during critical windows of early brain development. Human trials and cohort studies show more heterogeneous results, with the clearest benefits emerging in populations with low baseline PUFA status, learning difficulties, ADHD, or preterm birth. This heterogeneity is compounded by considerable variability in dosage, intervention duration and timing, and outcome measures across studies, as detailed in Section 2.4. Individual and ethnic variation in the main genes involved in PUFA metabolism further affects individual responsiveness to PUFA intake and supplementation. This highlights the need for more population-specific research, an area in which Mediterranean populations, including Lebanon, remain underrepresented. Despite this gap, Lebanon’s Mediterranean diet already offers a practical way to improve PUFA and MUFA intake among pregnant women, children, and adolescents. The challenge is improving adherence through nutrition education, dietary guidelines, and public health policy. Future research should prioritize studies in the MENA region examining PUFA-gene interactions and their relationship with neurodevelopmental outcomes, to allow tailored, evidence-based dietary recommendations for this population.

## Figures and Tables

**Table 2:** Effects of Omega-3 PUFAs on Cognition in Children and Adolescents.

Study (Year)	Study Design	Country	Age Group (*n*)	Clinical Group	Intervention	Findings	Limitations
Milte et al., 2012 [42]	RCT	Australia	Children (7–12 years) *n* = 90)	ADHD	EPA-rich oil (EPA 1109 mg + DHA 108 mg/day) vs. DHA-rich oil (EPA 264 mg + DHA 1032 mg/day) vs. safflower oil (control) for 16 weeks	Beneficial (specific subgroup and domain)—no significant group differences in literacy, cognition, or behavior; increased erythrocyte DHA (4 mo) correlated with improved word reading and lower oppositional behavior—effects stronger in children with comorbid learning difficulties	Reduced statistical power; ~50% of sample lacked formal ADHD diagnosis; treatment period.
Chang et al., 2019 [43]	RCT, 12 weeks	Taiwan	Children/adolescents (6–18 years) *n* = 92	ADHD	High-dose EPA (1.2 g/day) vs. placebo, for 12 weeks	Beneficial (specific subgroup)—beneficial effect on cognitive symptoms only in children with low baseline EPA; negative effect in those with high baseline EPA, highlighting the importance of assessing blood PUFA profile before supplementation	Short treatment period; mixed group of ADHD children; judged results by effect size rather than by statistical significance
Matsudaira et al., 2015 [44]	RCT	UK	Adolescents (males 12–16 years) *n* = 76	ADHD	Omega-3 and omega-6 supplementation, 6 capsules/day (EPA 558 mg + DHA 174 mg + GLA 60 mg + vit E 9.6 mg), for 12 weeks	No benefit—no effect on ADHD symptoms	Underpowered; short duration; low dose; high dropout; inconsistent teacher-rated ADHD symptom scores (due to teacher turnover), male-only sample.
Pinar-Martí et al., 2023 [21]	Cross-sectional	Spain	Adolescents (13.8 ± 0.9 years) *n* = 372	Typically developing	Red blood cell DHA and ALA levels (observational)	Positive association—higher DHA associated with better attention (faster reaction time, lower executive conflict); ALA associated with lower impulsivity but not consistently with attention performance	Cross-sectional (no causality); unmeasured confounders possible; reduced sample size from missing data.
Morandini et al., 2022 [45]	Neuroimaging study	USA (Healthy Brain Network)	Children/adolescents (7–17 years) *n* = 24	Neurotypical	Dietary omega-3 intake (observational)	No association—vigilant attention did not differ by dietary omega-3 intake	Cross-sectional (no causality); small sample size; reliance on self-reported dietary recall data instead of biomarkers.
Richardson et al., 2012 (DOLAB) [46]	RCT	UK	Children (7–9 years) *n* = 362	Underperforming readers	DHA supplementation (600 mg/day), for 16 weeks	Beneficial (specific subgroup)—no effect on reading in full sample, but significant improvement in the pre-specified subgroup with lowest reading scores (≤20th centile)	Limited generalizability (only underperforming readers); incomplete compliance data; DHA alone, not combined with EPA; strong placebo effect on parent ratings.
Sittiprapaporn et al., 2022 [47]	RCT	Thailand	Children (6–12 years) *n* = 120	General/typically developing	Low-dose (260 mg DHA) vs. high-dose (520 mg DHA) DHA fish oil vs. placebo, for 12 weeks	Beneficial (specific domain)—increased ERP amplitudes (neurophysiological improvement) with a dose–response effect, but no significant differences in reaction time, accuracy, or error rates on behavioral cognitive tests	Short treatment period; behavioral tests possibly less sensitive than brain activity measures.
Al-Ghannami et al., 2019 [48]	RCT	Oman	Children (9–10 years) *n* = 132	General/typically developing	Fish oil supplement (403 mg DHA/day) vs. 100 g of grilled fish meal (no control group) for 12 weeks	Beneficial (specific domain)—fish oil outperformed fish meal on one of three cognitive tests (Trail Making Test)	No placebo/control group (open-label); unequal DHA doses between arms; unvalidated ADHD scale for local population; single-city sample.
Shahabi et al., 2025 (ECLIPSES cohort) [49]	Cohort study	Spain	Pregnant women (12 and 36 weeks)/infants (54 days postpartum) *n* = 336 mother–child pairs	General/community cohort	Maternal omega-6: omega-3 and ARA/DHA ratios (observational)	Adverse association—higher *n*-6/*n*-3 and ARA/DHA ratios in the third trimester (not first) were negatively associated with infant motor development;	Unmeasured psychosocial factors (e.g., parental mental health); observational design (no causality); short-term outcome assessment only
Sarrafi et al., 2024 [50]	RCT	Iran	Pregnant women/infants (9–12 months) *n* = 150 mothers, 144 infants	General/community cohort	Omega-3 supplementation (120 mg DHA + 180 mg EPA/day) vs. placebo from 21st week of pregnancy to 30 days postpartum	No benefit—no significant effect on neurodevelopment or growth	Low dose; low baseline fish intake population; parent-reported outcome tool only
Guillot et al., 2022 [51]	RCT	Canada	Infants (18–22 months) *n* = 528	Preterm birth	High dose of maternal DHA supplementation (1200 mg) vs. placebo from birth to 36 weeks postmenstrual age	Beneficial (specific subgroup and domain)—no overall improvement in neurodevelopmental outcomes. Potential language benefits in a subgroup born before 27 weeks’ gestation	No correction for multiple outcomes tested; high-socioeconomic status cohort limits generalizability; test tool may miss subtle/behavioral effects; some infants also received IV DHA (confounder).
Baack et al., 2015 [52]	Cross-sectional	USA	Neonates *n* = 90	Preterm vs. term infants	Blood LCPUFA levels measured within first 7 days of life (observational)	N/A (descriptive)—preterm infants had significantly lower DHA/ARA levels than term infants, correlating with gestational age	No maternal dietary/supplement data collected; single-center study in a low-fish-intake region, limiting generalizability.

Abbreviations: ADHD, attention-deficit/hyperactivity disorder; ALA, α-linolenic acid; ARA, arachidonic acid; DHA, docosahexaenoic acid; EPA, eicosapentaenoic acid; ERP, event-related potential; GLA, γ-linolenic acid; IV, intravenous; LCPUFA, long-chain polyunsaturated fatty acid; N/A, not applicable; *n*-3, omega-3; *n*-6, omega-6; PUFA, polyunsaturated fatty acid; RCT, randomized controlled trial.

## Data Availability

No new data were created in this study. Data sharing is not applicable to this article.

## Abbreviations

**Abbreviation**	**Definition**
ADHD	Attention-Deficit/Hyperactivity Disorder
ALA	Alpha-Linolenic Acid
Aqp4	Aquaporin-4
BBB	Blood-brain-barrier
D5D	Delta-5 Desaturase
D6D	Delta-6 Desaturase
DGLA	Dihomo-γ-Linolenic Acid
DHA	Docosahexaenoic Acid
EFSA	European Food Safety Authority
ELOVL	Elongation of Very Long chain fatty acids
EPA	Eicosapentaenoic Acid
ERP	Event-Related Potential
ETA	Eicosatetraenoic acid
FADS	Fatty Acid Desaturase
FAO	Food and Agriculture Organization
GLA	γ-linolenic acid
GWAS	Genome-Wide Association Study
HF	High-fat
HUFA	Highly Unsaturated Fatty Acids
IL-5	Interleukin-5
IL-6	Interleuki*n*-6
KC/GRO	keratinocyte chemoattractant/growth-regulated oncogene
LA	Linoleic Acid
LCPUFA	Long-Chain- Polyunsaturated Fatty Acid
LD	Linkage Disequilibrium
LF	Low-fat
MAPKAPK2	Mitogen-Activated Protein Kinase-Activated Protein Kinase-2
MUFA	Monounsaturated Fatty Acid
Omega-3	ω-3
PUFA	Polyunsaturated Fatty Acid
RCT	Randomized Controlled Trial
S100b	S100 calcium-binding protein B
SNP	Single Nucleotide Polymorphism
